# Effects of Thermal Oxidation and Proton Irradiation on Optically Detected Magnetic Resonance Sensitivity in Sub-100 nm Nanodiamonds

**DOI:** 10.1021/acsami.4c08780

**Published:** 2025-03-30

**Authors:** Pietro Aprà, Gabriele Zanelli, Elena Losero, Nour-Hanne Amine, Greta Andrini, Mario Barozzi, Ettore Bernardi, Adam Britel, Roberto Canteri, Ivo Pietro Degiovanni, Lorenzo Mino, Ekaterina Moreva, Paolo Olivero, Elisa Redolfi, Claudia Stella, Sofia Sturari, Paolo Traina, Veronica Varzi, Marco Genovese, Federico Picollo

**Affiliations:** † National Institute for Nuclear Physics (Section of Torino), Via P. Giuria 1, 10125 Torino, Italy; ‡ Istituto Nazionale di Ricerca Metrologica, Strada delle Cacce 91, 10135 Torino, Italy; § Physics Department, University of Torino, Via P. Giuria 1, 10125 Torino, Italy; ∥ NIS Inter-Departmental Centre, Via G. Quarello 15/a, 10135 Torino, Italy; ⊥ Center for Sensors and Devices, Bruno Kessler Foundation, Via Sommarive 18, Povo, I-38123 Trento, Italy; # Chemistry Department, University of Torino, Via P. Giuria 7, 10125 Torino, Italy; ¶ Politecnico di Torino, Corso Castelfidardo 39, 10129 Torino, Italy

**Keywords:** nanodiamonds, NV sensing, ODMR sensitivity, quantum sensing, proton irradiation

## Abstract

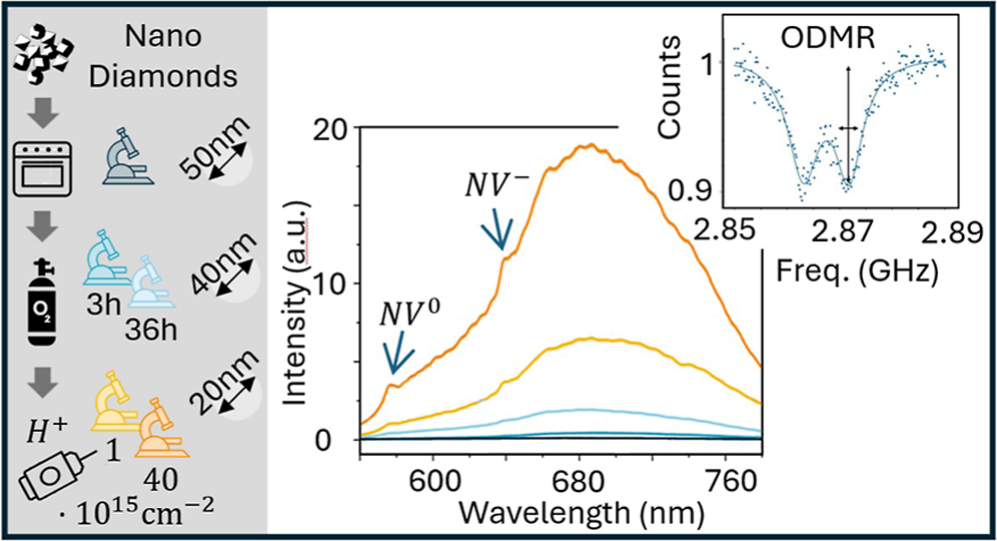

In recent decades, nanodiamonds (NDs) have emerged as innovative nanotools for weak magnetic fields and small temperature variation sensing, especially in biological systems. At the basis of the use of NDs as quantum sensors are nitrogen-vacancy center lattice defects, whose electronic structures are influenced by the surrounding environment and can be probed by the optically detected magnetic resonance technique. Ideally, limiting the NDs’ size as much as possible is important to ensure higher biocompatibility and provide higher spatial resolution. However, size reduction typically worsens the NDs’ sensing properties. This study endeavors to obtain sub-100 nm NDs suitable to be used as quantum sensors. Thermal processing and surface oxidations were performed to purify NDs and control their surface chemistry and size. Ion irradiation techniques were also employed to increase the concentration of the nitrogen-vacancy centers. The impact of these processes was explored in terms of surface chemistry (diffuse reflectance infrared Fourier transform spectroscopy), structural and optical properties (Raman and photoluminescence spectroscopy), dimension variation (atomic force microscopy measurements), and optically detected magnetic resonance temperature sensitivity. Our results demonstrate how surface optimization and defect density enhancement can reduce the detrimental impact of size reduction, opening to the possibility of minimally invasive high-performance sensing of physical quantities in biological environments with nanoscale spatial resolution.

## 1 Introduction

Currently, nanodiamonds (NDs) are attracting increasing interest in the context of biomedical sensing, thanks to the exploitation of optically active photoluminescent centers that can be incorporated into their crystal lattice.^1−4^ One of the most studied defects is the nitrogen-vacancy (NV) center. In its negatively charged state, it has been proposed and widely explored as a sensor for temperature variations and magnetic fields, based on the optically detected magnetic resonance (ODMR) technique.^5−10^ The small size of NDs and their chemical inertness and biocompatibility^2,6,11−13^ allow their interfacing with biological samples without altering cellular functioning. Previous experiments already demonstrated the effectiveness of this technique for intracellular sensing of temperature variations both in vitro and in vivo,^14−16^ reaching sensitivities as low as 0.6 . This allows observing the thermal effects induced by chemical stimuli and metabolic activity alteration,^17^ a line of work that culminated with measuring the effect on cell temperature of the excitation of neuron activity.^15^ Temperature gradient mapping of single cells was also demonstrated.^15,18−20^

Although the technique is showing increasing success, one of the main limitations resides in the weak and unstable fluorescence of NV^–^ centers in small nanocrystals (5–10 nm). This is due to the limited number of NVs that can form within the small volume of the nanoparticle and to their destabilization caused by the interaction with the nearby particle surface, which often induces photoionization effects.^16^ Therefore, most of the publications reporting ODMR sensing implementation in biological systems employ NDs as large as 100 nm or more^14,17,21−23^ which is suboptimal for certain biological applications, especially in the perspective of in vivo measurements.^24,25^ Indeed, the benefits of smaller NDs can reside in improved drug delivery efficiency by providing a larger relative surface area, enabling the loading of substantial drug quantities onto the surface. Small particles are also advantageous for traversing biological barriers and aiding in the clearance. Furthermore, a smaller size would allow to possibly reduce the distance from the system of interest: together with proper surface functionalization, small NDs would allow the measurement of physical quantities (e.g., steep temperature gradients and weak magnetic fields) at specifically targeted locations inside a cell, at the nanometric level. For example, while considering cellular membrane channels, presenting dimensions on the order of a few tens of nanometers, using NDs of comparable size is of the utmost importance for getting truly representative results. A narrow size distribution is also preferred, as it ensures consistent behavior among particles, enhancing data reproducibility.

According to the Stokes–Einstein equation, smaller ND particles are expected to have higher diffusion coefficient than bigger ones in a liquid environment, as is typical in biological applications.^26^ This could represent a limiting factor in terms of sensing performance; however, different mitigation strategies can be adopted. Aside from optimizing the sample preparation strategy (for example, in terms of NDs’ incubation time), both NDs’ functionalization and tracking can be considered. Functionalization allows the ND to bond to a specific site, thus presenting two advantages at the same time: the investigation of specific cell sites and random movement reduction. NDs’ tracking is based on close-range scans around the initial position, followed by movement of the scanning hardware to the new optimal position. Tracking the NDs’ position and rotation in cellular medium in real time has already been implemented in several works.^17,27,28^

Consequently, in this context, the assessment of processing procedures aimed at simultaneously reducing NDs size without losing performance in terms of ODMR sensitivity is of relevant interest. Indeed, as reported in several works and reviewed in ref (29), NVs’ sensing performances typically decreases while decreasing their distance from the surface.

To this scope, the tuning of surface properties and the selection of proper irradiation parameters for the control of NV centers’ charge state and density are expected to have a significant impact.

As a result of their synthesis process, the surface of pristine NDs is generally affected by a significant amount of disordered sp^2^ carbon phases, with a detrimental impact on the optical properties of the NV centers present in the diamond core. Purification processes have been developed in previous works, based both on chemical and thermal oxidation.^30−34^ These treatments also play a crucial role in determining the surface terminations, which are demonstrated to strongly impact the NV centers’ charge state distribution.^35^ Optimizing this distribution is crucial since, ideally, only the negatively charged centers (NV^–^) should be present in the nanoparticle, while the neutral centers (NV^0^) represent a source of background fluorescence noise. Moreover, the same processes have been shown to be effective in reducing the NDs’ size distribution, when performed in highly aggressive conditions, thus allowing simultaneous size reduction and NV charge state stabilization. Furthermore, since enhancing the NV concentration allows improving the signal-to-noise ratio and therefore the NDs’ sensing properties, the creation of additional NV centers is explored. This can be achieved, among other techniques, by means of proton irradiation.^36,37^ Following proton irradiation, a high-temperature (i.e., ∼800 °C) thermal annealing in an inert atmosphere is required to allow the proton-induced vacancies to get coupled with the nitrogen impurities, which are typically present with the concentration order of 100 ppm in NDs obtained from the fragmentation of crystals synthesized with high-pressure high-temperature (HPHT) technique (IIb type).^38,39^

In this work, we consider a combination of thermal oxidation and proton irradiation processes in order to optimize the quantum sensing properties of small NDs. Size reduction is achieved by performing prolonged thermal air oxidation, which also determines the increase in surface oxygen-containing chemical groups. Indeed, the latter is demonstrated to favor NV^–^ centers with respect to NV^0^,^35,40,41^ thus potentially counterbalancing, together with proton-beam-induced NV density increase, the detrimental size reduction effect on their sensing properties. Processed samples were then characterized with photoluminescence (PL) and diffuse reflectance infrared Fourier transform (DRIFT) spectroscopies to assess the process’ effect in terms of both optophysical and chemical properties, together with dynamic light scattering (DLS) analysis to evaluate the nanoparticles’ dispersibility in solution. Atomic force microscopy (AFM) measurements were performed to measure the size of the NDs after the described treatments. Finally, the temperature sensitivity was estimated in ODMR measurements to assess the effect of the processes on the technique performance.

## 2 Experimental Section

### 2.1 Annealing and Oxidation

Commercial NDs produced from fragmentation of HPHT diamond (Pureon MSY 0-0.1) and presenting a size ranging from a few nanometers to up to 100 nm, with a median size of ∼55 nm were used. High-temperature thermal annealing at 800 °C for 2 h in N_2_ flow was initially performed to convert to graphite the amorphous carbon phases covering the NDs’ surface (the resulting NDs are referred as “AnnND” in the following). Subsequently, surface oxidation was performed by means of thermal etching in an air environment at 500 °C. Additional information about the process and the selection of these conditions can be found in previous works.^34,42^ Two process durations were explored, namely 3 and 36 h (labeled “Ox^low^ND” and “Ox^high^ND”, respectively) in a tubular furnace. Both these preirradiation annealing and oxidation thermal processes were carried out on NDs in powder form contained in alumina crucibles.

### 2.2 Proton Irradiation

A fraction of the Ox^high^NDs was irradiated with a proton beam to create lattice vacancies, which can subsequently combine with native nitrogen (∼100 ppm concentration) to form additional NVs. Following dispersion in isopropyl alcohol, NDs were deposited on a Si substrate and dried, thus creating an ∼(30 ± 10) μm thick layer. This thickness ensures a flat damage profile along the whole layer during ion irradiation (avoiding the Bragg peak effect, see Figure S1 from SRIM simulation of the linear vacancy density of 2 MeV protons in NDs). A 4 × 4 mm^2^ 2 MeV H^+^ ion beam at the AN2000 accelerator facility of the INFN National Laboratories of Legnaro was employed, with a beam current varying in the 0.8–1 μA range. Two fluences were selected, namely, 1 × 10^15^ and 4 × 10^16^ cm^–2^, estimated from the integrated charge collected from the irradiation chamber. To evaluate the irradiation damage profile, a Monte Carlo simulation was carried out using SRIM software,^43^ by setting a displacement energy of 50 eV and a density of 1.5 g cm^–3^, estimated by weighting a known volume of compacted NDs. The simulations confirmed that 2 MeV protons deliver an almost constant damage density across the whole thickness of the NDs’ layer deposited on the silicon substrate. Following ion irradiation, 800 °C thermal annealing in a N_2_ flow was performed for 4 h to promote the formation of NV centers by allowing the generated vacancies to get coupled with the native N impurities. Since the latter process is chemically reducing, the introduction of oxygen-containing species on the surface was obtained by further oxidizing NDs for 12 h at 500 °C, with an additional effect in terms of size reduction. The resulting samples are referred as “Irr^low^ND” and “Irr^high^ND”, respectively, in the cases of 1 × 10^15^ cm^–2^ and 4 × 10^16^ cm^–2^ irradiation fluences, respectively. As estimated with SRIM code, these two fluences correspond to vacancy density values of ∼1 × 10^18^ cm^–3^ (∼5 ppm) and 4 × 10^19^ cm^–3^ (∼200 ppm), respectively.

**Figure 1:**
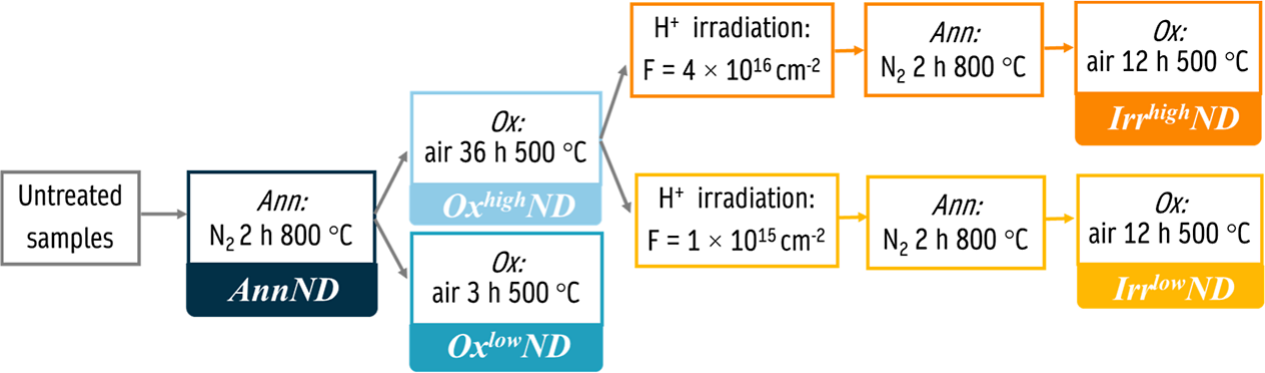
Summary of the different samples compared in this work. The main parameters of the processes are reported.

The processes carried out on the different samples are summarized in Figure 1.

### 2.3 DRIFT Spectroscopy

In order to gain insight into the NDs’ surface chemistry following thermal treatments, DRIFT spectroscopy was performed on Ann-ND and Ox^high/low^ND samples. Spectra were acquired with a Bruker Equinox 55 FTIR spectrometer, equipped with a mercury–cadmium–telluride (MCT) cryogenic detector; 64 interferograms (recorded at 2 cm^–1^ resolution) were averaged for each spectrum. The reflectance data were successively converted in pseudoabsorbance: *A* = −log *R*, where *R* is the measured reflectance.

### 2.4 DLS Analysis

Assessing the level of aggregation and stability of NDs in a liquid environment is very relevant for biological applications. Therefore, samples were analyzed in this respect via DLS measurements. We exploited a Zetasizer Nano from Malvern Instruments ZS (Malvern, UK) operated with a 4 mW He–Ne generator at 633 nm laser (scattering angle 173° for the typical diamond refractive index, i.e., 2.4). We carried out size analysis in 0.5 mM NaCl aqueous solution with an ND concentration of 50 μg/mL. The suspensions were prepared with annealed (AnnND) and annealed + oxidized (Ox^low/high^ND) NDs. Before any acquisition, the solutions were sonicated for 15 min.

### 2.5 Raman and PL Spectroscopy

Raman and PL spectra of Ox^low/high^ND and Irr^low/high^ND samples, deposited on a ∼(30 ± 10) μm thick layer, were acquired with a Horiba Jobin Yvon HR800 Raman microspectrometer equipped with a continuous NdYAG 532 nm excitation laser, focused with a 20× air objective, and a CCD detection system with a Peltier cooling system (−70 °C). A spatial resolution of ∼2 μm in diameter and ∼16 μm in confocal depth was feasible with the employed objective. 600 lines mm^–1^ diffraction grating was employed, guaranteeing a spectral resolution of ∼3 cm^–1^.

### 2.6 Atomic Force Microscopy

AFM measurements were performed using an NX20 AFM Park system, in the noncontact mode, which allows noninvasive and highly accurate topography imaging while maintaining tip and sample preservation. Isolated NDs were deposited and dried on Si substrates, and several AFM maps were acquired in order to measure a statistically significant number of NDs (more than 300 for each sample). Each map scans over a (10 × 10) μm^2^ area, with a resolution of 1024 pixels × 1024 pixels. The acquired images were further processed using open software Gwyddion. The procedure included image flattening, isolated NDs’ identification, and collection of specific parameters for each identified ND. For completeness, an example of the image processing is reported in the Supporting Information, see Figure S3.

### 2.7 PL and ODMR Setup

**Figure 2:**
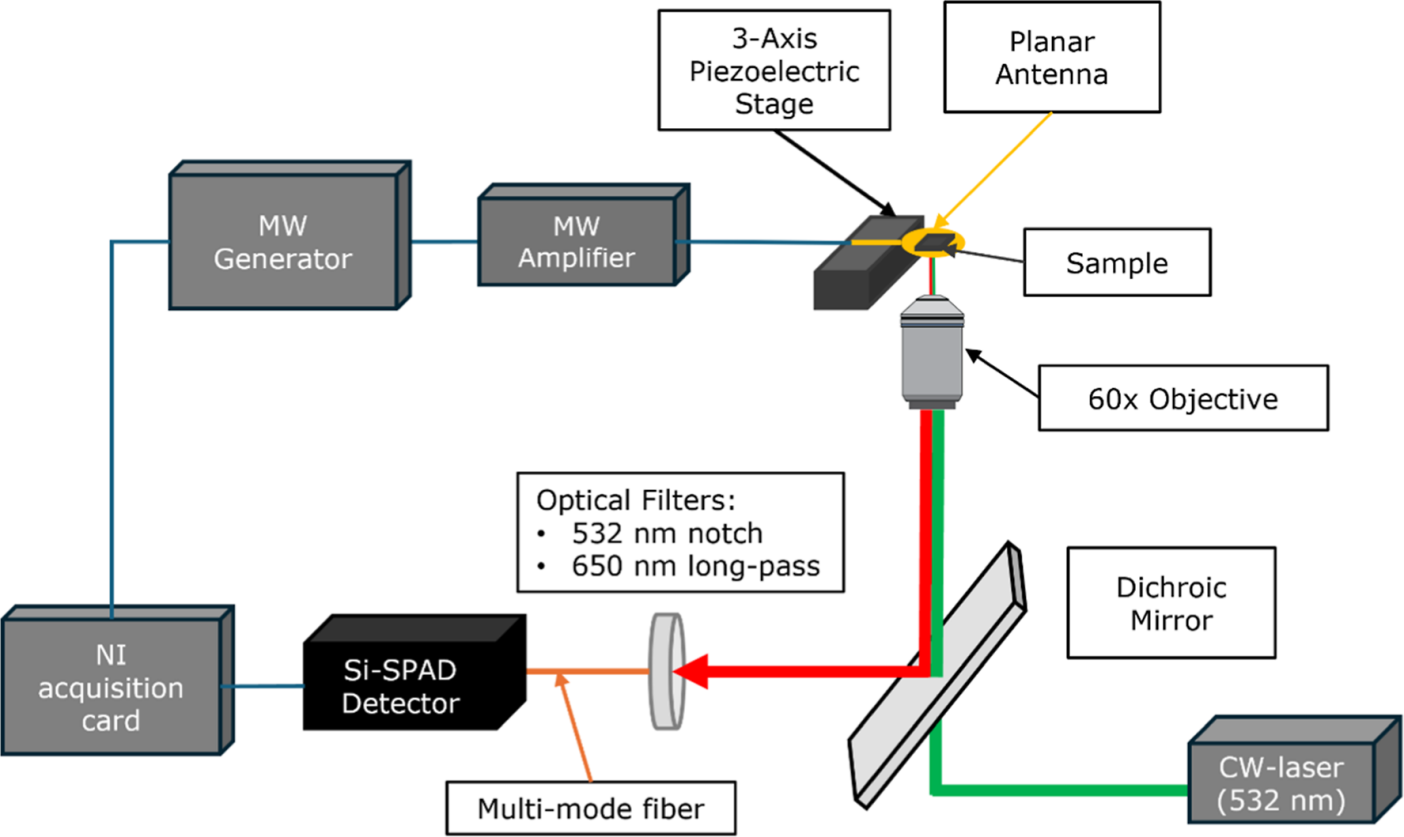
Schematic of the confocal microscope setup used for measuring PL and ODMR spectrum from isolated NDs.

To perform PL measurements and ODMR on isolated NDs, a home-built single-photon confocal microscope system was employed (Figure 2). To perform these measurements, NDs were diluted in water, deposited, and dried on Si substrates. As for AFM, the solution was prepared at the highest concentration, allowing, once deposited, the observation of isolated NDs. Correlative mapping,^44^ between AFM and PL maps, could be an alternative approach which can be explored in future works. The sample was mounted on a 10 nm resolution piezoelectric stage. The excitation light (100 μW optical power, low enough to be compatible with biological experiments) at 532 nm was focused on the NDs through an air objective (60×, NA = 0.9, optical spatial resolution ∼400 nm). The PL signal was collected by the same objective, spectrally filtered by a notch filter centered at the laser wavelength plus a long-pass filter (at 650 nm), fiber coupled, and finally sent to the detector, consisting of a silicon single-photon-sensitive avalanche detector (SPAD). Further details about PL maps acquisition and processing can be found in the Supporting Information, see Figure S4.

To perform ODMR measurements, a commercial microwave (MW) generator (Keysight N5183B) coupled with a specifically designed planar antenna^45^ allowed to uniformly address the NDs in the region of interest. In order to find the optimal sensing conditions, both laser power and MW power were systematically varied and the resulting ODMR spectrum compared: it turned out that the same sensing conditions may be used for all the samples under consideration. More details on the setup developed in our laboratory can be found in ref (15).

## 3 Results and Discussion

### 3.1 Surface Chemistry and Hydrophilicity Characterization

**Figure 3:**
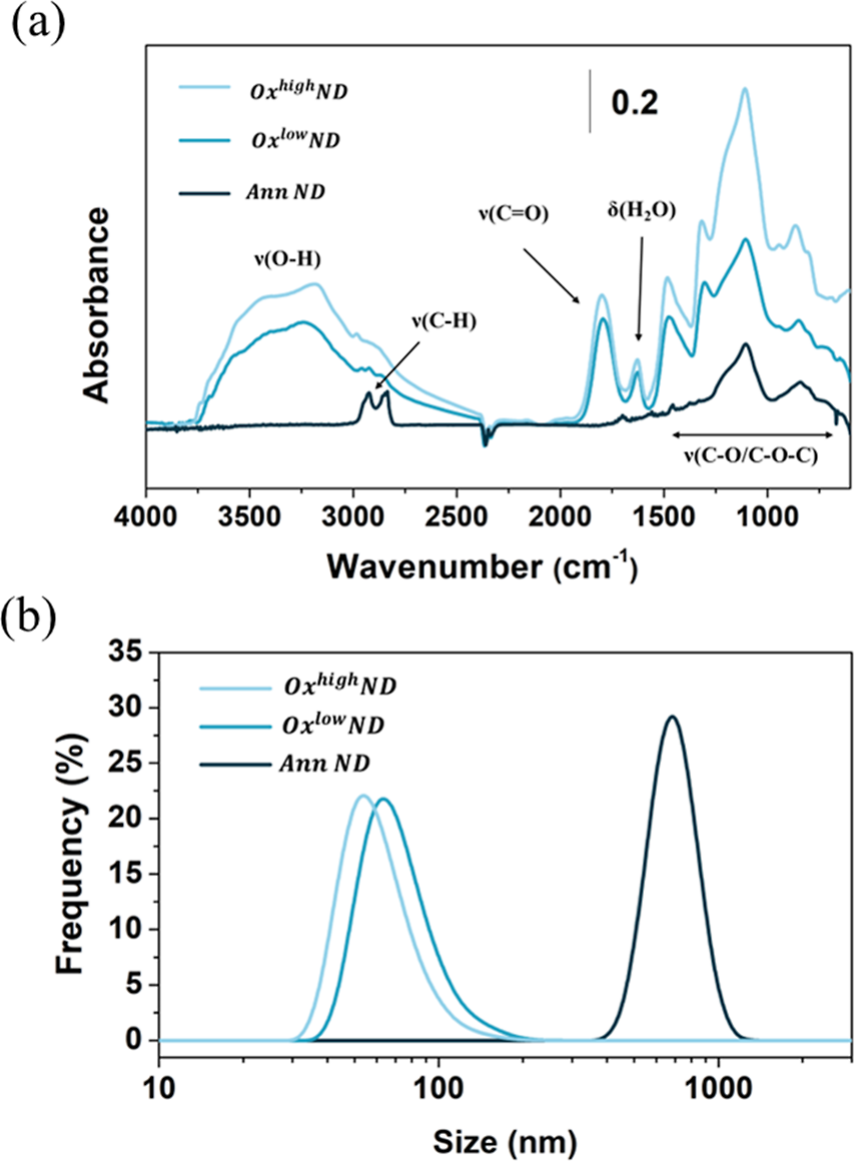
(a) DRIFT spectra of differently oxidized NDs in comparison with annealed NDs and (b) size of NDs in water solution assessed by DLS measurements.

The oxidation process has an important impact on the NDs’ surface termination. This is demonstrated by comparing the DRIFT spectra before and after the purification process via oxidation, as reported in Figure 3a.

Spectra following oxidation show the appearance, proportionally with the process time, of new IR bands, typical of oxygenated surface terminations, including ν(C=O) at 1780 cm^–1^ and different ν(C–O) vibrations in the 1300–1000 cm^–1^ range, which can be associated with different oxygen-containing moieties present in carboxylic acids, esters, lactones, and acid anhydrides.^34,42,46^ Moreover, ν(O–H) modes in the 3700–3000 cm^–1^ spectral region and the H_2_O bending at 1630 cm^–1^ become evident because of water adsorption from environmental moisture. Indeed, in a previous work,^34^ this was demonstrated to be associated with a higher hydrophilicity induced by the presence of oxygen-containing groups. The same work also showed how by repeating DRIFT measurement upon simultaneous thermal heating up to 400 °C, H_2_O disappears between 100 and 200 °C. This is enforced by the results provided by DLS measurements (Figure 3b), which show a significant improvement in terms of stability and dispersibility in water-based solution by oxidized NDs. Conversely, a higher size is detected in the case of AnnND samples, whose surface is mainly covered by C–H terminations (see Figure 3a), showing a hydrophobic behavior and thus a higher aggregation level. Similar results are obtained by performing the same measurement in phosphate-buffered saline (see Figure S2 in the Supporting Information), guaranteeing proper dispersibility also for in vitro biological media.

### 3.2 Raman/PL Characterization

**Figure 4:**
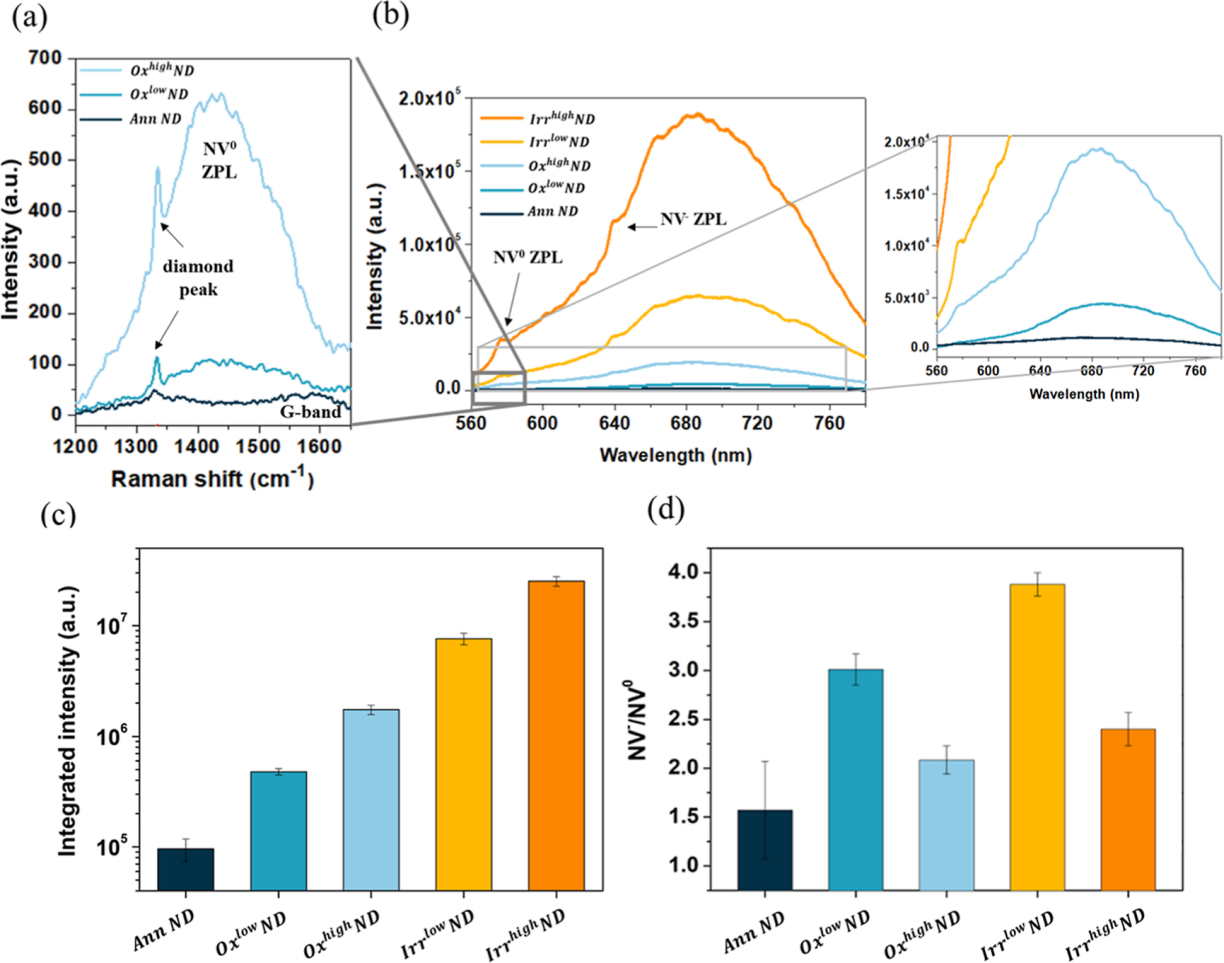
(a) Raman spectra of annealed and annealed + oxidized NDs after PL baseline subtraction weak G-band at 1580 cm^–1^ is observable for annealed NDs, which is then completely removed upon both oxidation conditions. Conversely, a clear increase of NV^0^ zero-phonon line is evident in the spectra. (b) PL spectra of annealed, annealed + oxidized NDs, and ion irradiated NDs (postannealing). (c) Integral of NV centers PL intensity and (d) NV^–^/NV^0^ ratio for the different processes.

Structural and fluorescence properties of NDs upon oxidation and ion irradiation processes are assessed via Raman and PL spectroscopy. Figure 4a shows the resulting Raman spectra for AnnND and Ox^low/high^ND samples after proper background subtraction, while Figure 4b reports PL spectra of AnnND, Ox^low/high^ND, and Irr^low/high^ND samples. The Raman spectrum collected from AnnNDs presents a G-band at ∼1580 cm^–1^, which is a feature that can be associated with the presence of disordered sp^2^ carbon phases on the surface of the nanoparticles. Indeed, following purification via air oxidation, the G-band disappears due to the selective etching of graphitic phases from the NDs’ surface, and the first-order Raman diamond peak around ∼1332 cm^–1^ becomes more evident. The PL spectra collected from Irr^low/high^ND samples show a wide fluorescence between 580 and 780 nm, arising from NV centers. NV^0^ and NV^–^ zero-phonon lines are evident at 576 and 638 nm, respectively.

Figure 4c reports the results of the spectral integration of the PL signals between 650 and 780 nm (consistently with filters employed for confocal PL analysis that is reported in Section 3.4) as a function of the process. A clear PL intensity enhancement is observed upon air oxidation, consistently with the reduction of the quenching effect due to the defective outer layers.^47^ Samples which underwent ion irradiation (and subsequent thermal annealing to enhance the NV centers density) show a strong increase in fluorescence, especially for the higher explored fluence (14 times higher than Ox^high^ND), in accordance with a higher number of created defects.

The NV^–^/NV^0^ ratio is evaluated by fitting linear combinations of individual NV^0^ and NV^–^ spectra, extrapolated as described in ref (48) (see Figure 4d). Oxidized NDs show a higher ratio with respect to annealed NDs, in accordance with the effect of the dipole moment associated with oxygen-containing functional groups.^49^ Three hours of the oxidation process (Ox^low^ND) is more effective than a 36 h one (Ox^high^ND) in promoting NV^–^ centers’ formation, probably due to the formation of complex varieties of oxygenated moieties with lower dipole moment with respect to simple carboxylic groups, such as anhydrides and lactones.^49,50^ Similar behavior is observed for the irradiation fluence: samples irradiated at 1 × 10^15^ cm^–2^ show a higher NV^–^/NV^0^ ratio with respect to 4 × 10^16^ cm^–2^ (Irr^low/high^ND). Indeed, according to the literature,^38^ by increasing the irradiation fluence, a smaller NV^–^/NV^0^ ratio occurs due to the limited charge transfer from nitrogen impurities, which behave as electron donors.

### 3.3 AFM Analysis

Oxidation and irradiation processes also have an impact on the NDs’ dimension. To investigate this aspect, AFM analysis was systematically performed on both Ox^low/high^ND and Irr^high^ND samples. Among the different parameters that can be extracted, we focus on the maximum height measured in each ND since this is a value which well represents the ND dimensions.^51^ The lateral dimensions, on the other hand, do not represent a good estimate since, given the NDs’ small dimensions, the shape of the tip plays an important role and would lead to an overestimation of the ND dimension.

**Figure 5:**
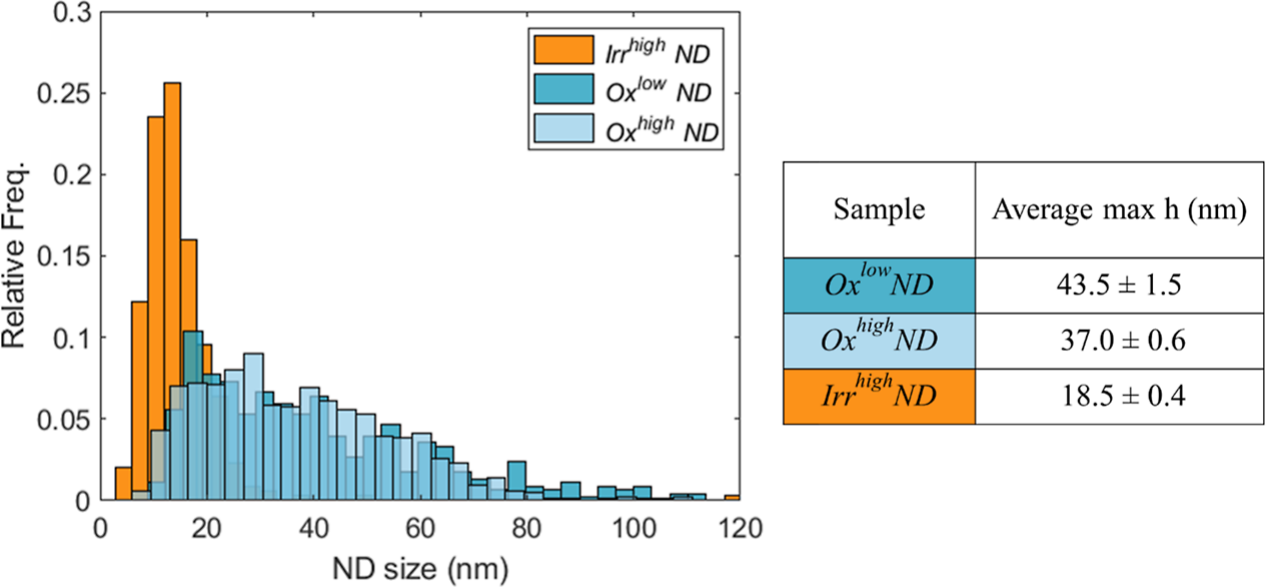
Normalized histograms reporting the diameter distribution for different ND samples (Irr^high^ND, Ox^high^ND, and Ox^low^ND), according to AFM analysis. The table reports the average size and associated standard deviation in the different cases.

The histograms reported in Figure 5 compare the different size distributions, and the average size results are reported in the same figure.

A slightly smaller size is observed in the case of Ox^high^ND samples with respect to Ox^low^ND samples, in accordance with the longer duration of the oxidation process. This demonstrates that the process affects not only the superficial sp^2^ carbon atoms but also the diamond core. Irr^high^ND samples show a relevant size reduction: this is well expected since the thermal annealing necessary to activate NV centers following ion irradiation induces surface graphitization, which is then removed by the further oxidation process. Similar results are expected in Irr^low^ND samples, which underwent analogous annealing and oxidation processes.

### 3.4 PL Analysis on Dispersed NDs

In view of ND-based sensing applications, it is important to test the NDs’ performance in a realistic experimental setting where isolated NDs are typically interrogated. While PL measurements shown in Figure 4 were conducted on thick layers of compacted NDs to obtain information concerning the structural and fluorescence properties of the overall diamond component, in real application scenarios, the PL analysis is conducted on single NDs or small aggregates (presenting dimensions below optical resolution), thus size distribution differences among samples are expected to have an impact on the outcome of the measurement.

**Figure 6:**
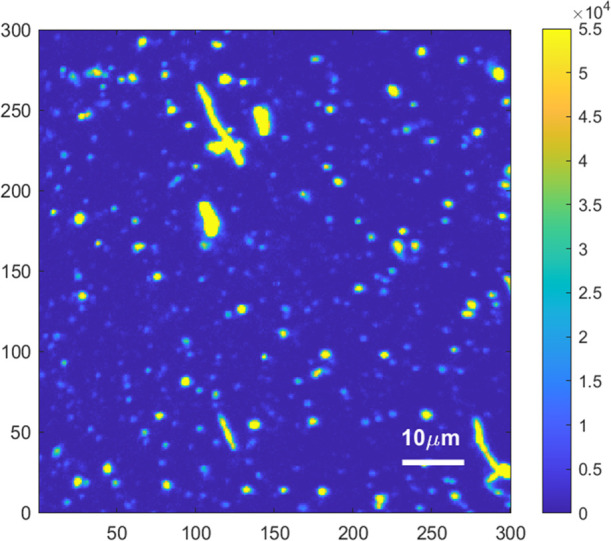
PL map acquired at our home-built scanning confocal setup for the Ox^low^ND sample. The area measures (80 × 80) μm^2^ scanned with a resolution of (300 × 300) pixels.

To collect significant statistics, we used a scanning confocal microscope (described in 2.7) on (80 × 80) μm^2^ regions, acquiring PL maps containing several hundred (i.e., ≥300) isolated NDs. An example is reported in Figure 6.

The raw PL maps are postprocessed to remove the background light and to automatically identify isolated NDs. For each ND, the maximum PL value is extracted. The average maximum values, for the 4 cases considered, are reported in Table 1.

**Table 1:**
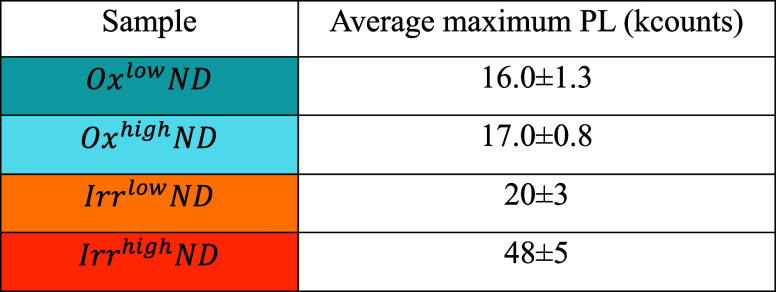
Average Maximum PL from Single NDs in the Different Samples

The Irr^high^ND samples, which underwent the highest dose irradiation process, present an average maximum intensity which is roughly 3 times higher with respect to Ox^high^ND ones.

This result is in apparent contrast with the PL enhancement reported in Figure 4c, where the PL from Irr^high^ND samples is 14 times higher than that in the Ox^high^ND ones. This can be explained considering that the PL measurement techniques are investigating ND samples dispersed with a different geometry over the sample holder. In fact, PL measured with the Raman microspectrometer was acquired by focusing the laser spot on a 30 μm thick uniform layer of compacted NDs (see Figure S5, top line); therefore, the number of measured NV centers (and thus the PL) depends on the excitation volume and on the NV density but not on the single ND dimension. On the other side, PL measured with the ODMR confocal setup arises from a single ND, or small aggregates below spatial resolution of the optical system (see Figure S5, bottom line). Therefore, in this case the PL depends on the NV density and on the single ND dimension. As a result, by comparing Ox^high^ND and Irr^high^ND samples, we observe a smaller PL enhancement when we measure PL from single NDs rather than from a compacted NDs’ layer. From AFM analysis, we know that the diameter of Irr^high^NDs is roughly half than that of the Ox^high^ND ones, which corresponds to a volume reduction factor γ = 0.5^3^ (0.125). Thus, we would expect this reduction factor in the PL enhancement, i.e., PL(Irr_dispersed_^high^ND) ∼ PL(Ox_dispersed_^high^ND) ×  × γ.

This corresponds to an estimated PL equal to  kcounts ∼ 30 kcounts for the Irr^high^ND sample. The observed value is ∼48 kcounts (see Table 1): this discrepancy can probably be attributed to the fact that small aggregates, below spatial resolution of the optical system, contribute with higher fluorescence but are excluded in AFM analysis. Therefore, also considering that NDs are far from being spherical, assuming the volume reduction factor to be 0.5^3^ is a rough approximation.

**Figure 7:**
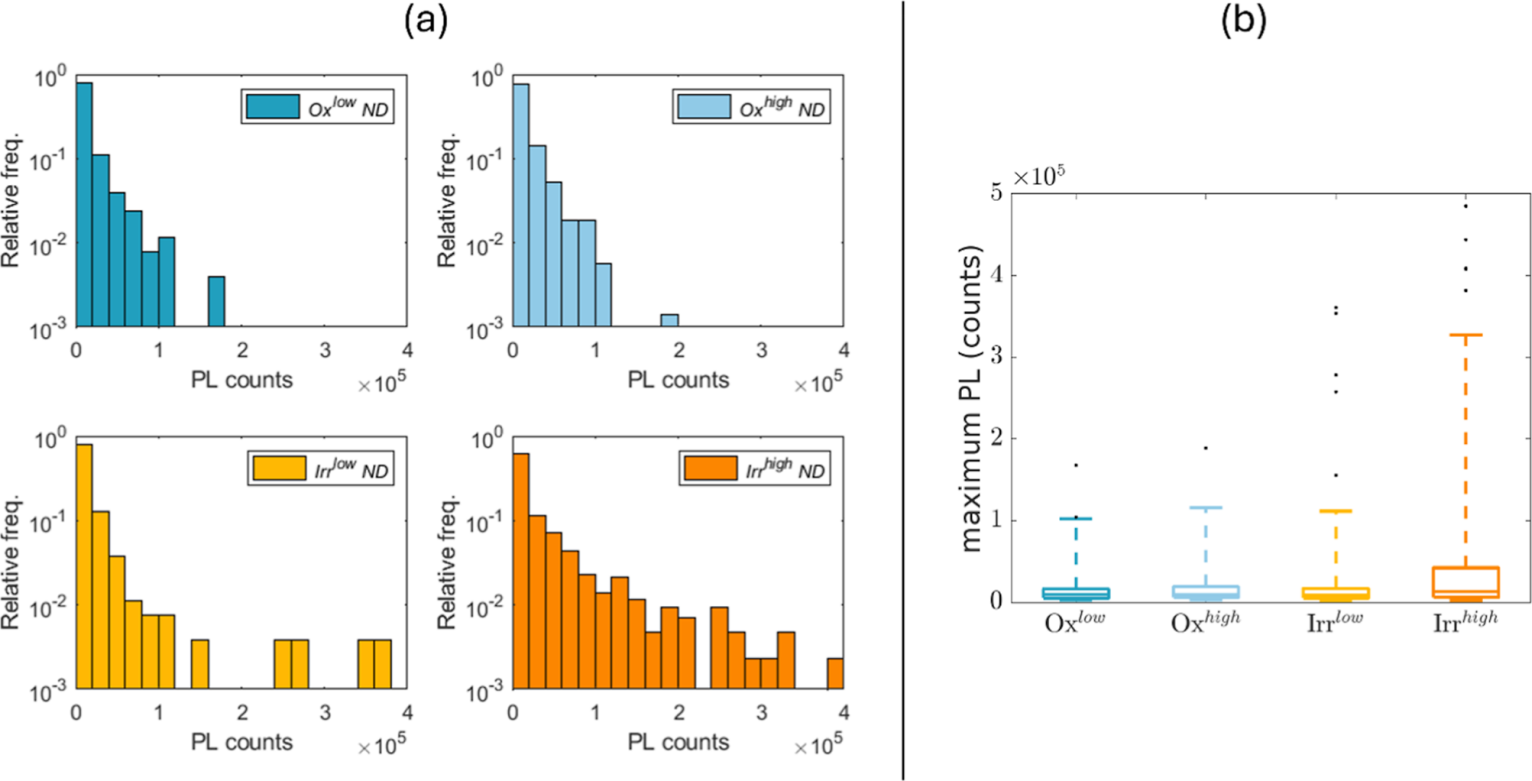
(a) Normalized histograms reporting the maximum PL distribution in the different ND samples. (b) Box and whisker plot of the PL counts in the different NDs samples. Data are obtained from isolated NDs (>300 in each case), identified on PL maps (as the one reported in Figure 6) acquired at our home-built scanning confocal setup.

In Figure 7a, we report the normalized frequency histograms of the maximum PL intensity in the different samples. Figure 7b shows the corresponding box and whisker plot.

In order to assess differences between the obtained distributions, they are compared by using the two-dimensional Kolmogorov–Smirnov test. Considering a significance level of 5%, it can be observed that the distribution for the Irr^high^ND samples is significantly different from the other three distributions. On the other hand, the distributions for the Ox^low/high^ND and Irr^low^ND samples are mutually compatible. The *p*-values of the hypothesis tests are reported in the Supporting Information (see Tables S1–S4).

**Figure 8:**
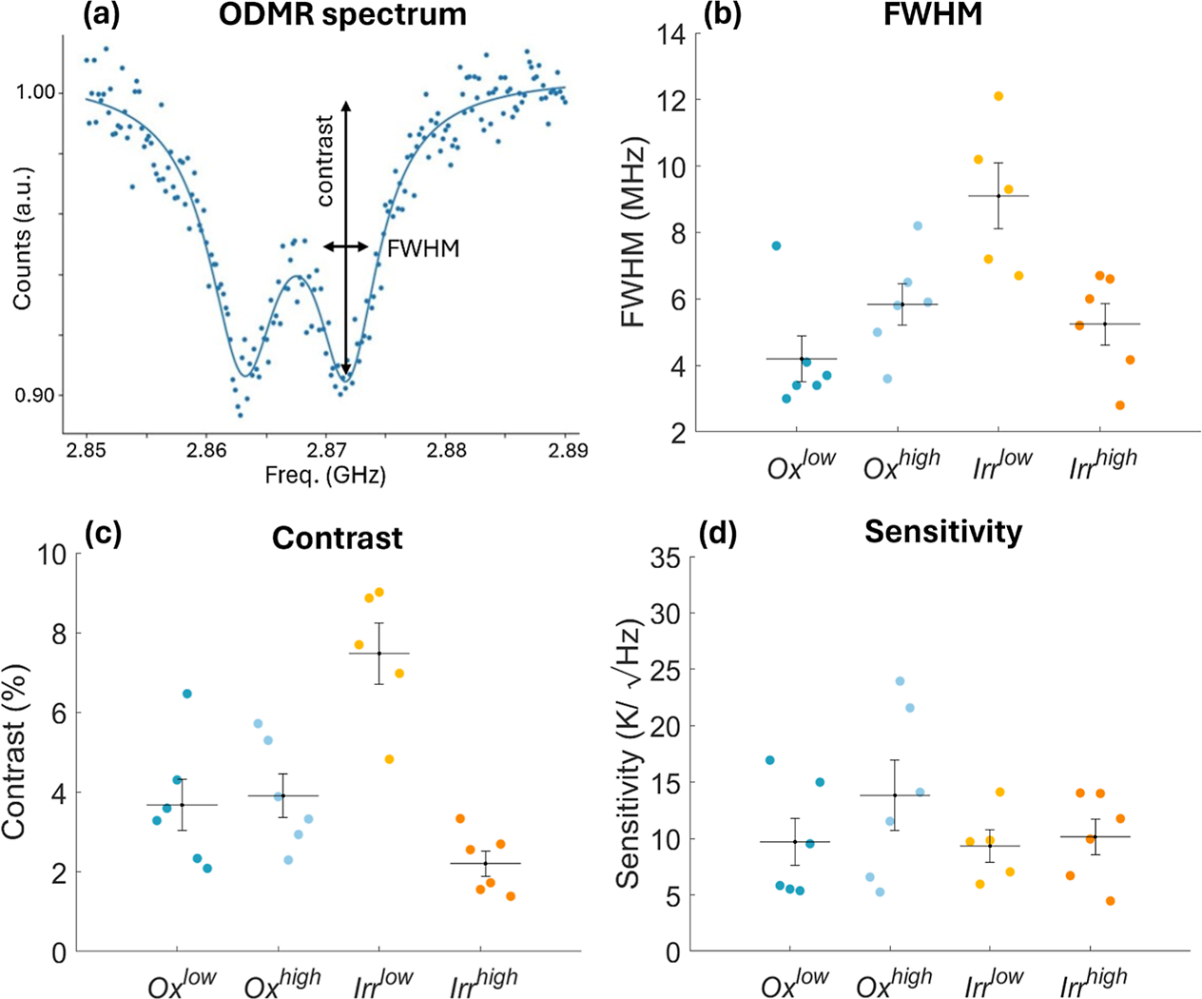
(a) Example of ODMR spectrum from an Irr^high^ND, normalized and fitted with a double Lorentzian curve. (b) fwhm, (c) contrast, and (d) shot-noise limited sensitivity (according to eq 1) for several NDs in the different samples.

The differences among the PL distributions can be appreciated also from the box and whisker plots, reported in Figure 8b. From this plot, the presence of isolated Irr^high^NDs presenting a much higher PL respect with the median value is clear.

### 3.5 ODMR Sensitivity

Finally, ODMR measurements are performed on several NDs from different sample batches in order to assess how the NDs’ processing affects their potential exploitation as quantum sensors. This set of measurements, performed on single NDs using our confocal setup, is crucial, since it allows assessing the NDs’ performances in a typical experimental configuration.

The continuous-wave ODMR protocol consists in simultaneously applying a variable frequency microwave field and nonresonant laser radiation to the selected ND while acquiring its emitted PL intensity.^52,53^ Thanks to the spin-dependent PL, this technique allows inspecting the ground-state electronic level structure of the NV centers.^54−56^ Being this structure-dependent on temperature^52,53^ (as well as electromagnetic fields), NVs in NDs can be used as temperature sensors.

For each ND sample under consideration, ODMR data are acquired for 300 s, allowing an adequate signal-to-noise ratio, and fitted with a double Lorentzian curve. An ODMR fit example, for Irr^high^ND, also showing the relevant fit parameters, is reported in Figure 8a. The presence of two dips is due to the intrinsic strain that is particularly intense in NDs and that breaks the spin-state degeneracy.^57^ ODMR spectra from Irr^low^NDs and Ox^high,low^NDs are not shown but qualitatively present very similar features.

For each ND, ODMR data are fitted and both contrast and line width are extracted as fit parameters. Contrast is typically expressed as a percentage and quantifies the PL drop in correspondence to the deep. Results for the different batches are compared in Figure 8b,c.

Performing a Welch *t*-Test, we conclude that, with the significance of 5%, in the Irr^low^ND samples the contrast and line width values are statistically different with respect to the other samples. Irr^low^ND samples are characterized by a higher contrast but also a broader line width. The *p*-values of the hypothesis tests are reported in the Supporting Information. The higher contrast values observed in the Irr^low^ND samples can be ascribed to the higher NV^–^/NV^0^ ratio (see Figure 2), which results in low NV^0^ background noise. The higher fwhm observed in the Irr^low^ND sample with respect to the OxND ones can be ascribed to the NDs’ size reduction, which leads to higher surface spin noise. Further irradiating the sample (Irr^high^) decreases the number of substitutional nitrogen (P1 defects) decreases. This leads to fwhm reduction^56^ which in our irradiation conditions results in observing the Irr^high^ sample with the same fwhm as in the oxidized cases.

A temperature change induces a proportional shift in the ODMR spectrum: the shot-noise-limited temperature sensitivity, expressed in , can be evaluated according to eq 1
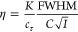
1where : temperature coupling constant, *D*_gs_: ground-state zero-field splitting, *T*: temperature, fwhm: spectral width, *C*: contrast. *I*: rate of detected photons, and *K*: parameter related to the specific profile of the spin resonance. For a Lorentzian profile, as assumed here, *K* ∼ 0.77.

This limit is discussed in several works among others^56,58−61^ and represents a lower limit for the sensitivity, while considering the continuous-wave ODMR measurement protocol for magnetic or temperature sensing (being the only difference in the value of the multiplicative coupling constant). Shot noise is fundamentally related to the Poissonian statistics of the PL counts and is unavoidable for any optical spin readout. This noise is dominating over the spin projection noise,^56^ thus representing the real lower bound in practice. Note that the shot-noise-limited sensitivity does not consider other noise sources which may be present in the setup, as for example electronic noise, laser-fluctuation noise, and environmental noise, however, various experimental works demonstrate a measured sensitivity in proximity with the independently evaluated shot-noise-limited one.^53,62,63^

The shot-noise-limited sensitivity results are reported in Figure 8d and quantify the potentialities of the different ND batches as temperature quantum sensors. In this work, we focus on temperature, being particularly interested in thermometric biological applications, but an equation analogous to eq 1 holds also for magnetic shot-noise sensitivity by replacing *c*_τ_ with the magnetic coupling factor. Sensitivity can be improved using more advanced ODMR protocols based on suitable sequences of microwave pulses (i.e., pulsed-ODMR^58^). Moreover, multimodal sensing (e.g., temperature and magnetic field fluctuations) is possible using the ODMR approach.

Although the improvements in terms of ODMR sensitivity are apparently negligible following the performed treatments, the relevance of these results must be considered in light of the substantially smaller size of the resulting NDs. Indeed, Irr^low/high^ND samples show the same sensitivity of unirradiated NDs, despite their diameter being reduced by more than 1/2 (and, consequently, the volume by more than 1/8). As a result, the protocol presented in this work demonstrates a viable opportunity and reproducible strategy to obtain sub-20 nm sized NDs without any sensitivity loss with respect to the initial, not irradiated, NDs. The absence of statistical differences between Irr^low^ND and Irr^high^ND samples’ sensitivities can be ascribed to the fact that although ion irradiation enhances the brightness of the Irr^high^ND samples, this effect is counterbalanced by the lower NV^–^/NV^0^ ratio observed with respect to Irr^low^ND samples, which results in a smaller contrast.

We also note that in the Irr^low/high^ND samples the sensitivity presents lower variance with respect with the Ox^low/high^ND ones. Together with the size variance reduction (see size distribution in Figure 5), this represents an advantage in experimental applications, since it facilitates the acquisition of homogeneous data from different NDs in the sample.

## 4 Conclusions

Size reduction in NDs is often performed in order to achieve nanocrystal dimensions that are suitable for specific biological applications and nanoscale metrology. Nonetheless, smaller NDs are generally associated with significantly worse performance in terms of optical properties and consequently as quantum sensors.^64^ In this work, we presented a protocol to obtain size reduction of NDs without affecting the ODMR sensitivity by means of thermal treatments and ion beam irradiation techniques. To the best of our knowledge, the obtained ODMR sensitivity is the best reported in the literature for NDs of these dimensions (∼20 nm diameter). The effect of oxidation and ion irradiation processes on NDs was explored in terms of surface chemistry, size, and optical properties. The first focus was to obtain NDs with higher NV^–^ density and to evaluate the impact on the ODMR sensitivity. Thermal oxidation proved to be effective in enhancing the optical properties and optimizing the NV^–^/NV^0^ ratio according to the induced surface chemistry, as demonstrated by DRIFT and Raman/PL spectroscopy performed on piled ND powders. Similarly, ion irradiation strongly enhanced fluorescence, especially at the higher fluences, while the NV^–^/NV^0^ ratio was found to be optimal at intermediate irradiation fluences (namely, 10^15^*p*^+^ cm^–2^). This observation reasonably explains the absence of significant differences between the ODMR sensitivities observed from Irr^low^ND and Irr^high^ND samples. Remarkably, upon ion irradiation and subsequent postprocessing, ODMR sensitivity was not degraded despite the ND size reduction induced by the ion irradiation post processing, as proved by AFM analysis. Further tests will be devoted to testing the process on different untreated NDs (in terms of both size and material properties), targeting samples presenting better initial sensitivity and thus potentially leading to better final sensitivity. In our work, we started from commercial HPHT NDs, which are industrially produced and thus easily available. However, their quantum performances are typically not optimal. On the other side, CVD NDs^65−67^ seem particularly promising, even if currently not routinely produced.

Typically, NDs used for intracellular temperature measurements are characterized by a diameter which is larger than 100–150 nm and a sensitivity of about 1–2  (see Table 1 in ref (21) for a comprehensive literature review). The dimension of the NDs explored in this work is significantly smaller, especially for the irradiated batches (Irr^low,high^ND), where the diameter is estimated to be <20 nm (see Figure 5). In this context, the achieved sensitivity of  (see Figure 8d) represents a fundamental step forward. NDs presenting such small dimensions, combined with proper functionalization, will allow intracellular temperature measurements with unprecedented spatial resolution (eventually enhanced by specific super-resolution methods^68^), e.g., allowing monitoring of cellular membrane channels’ (presenting a dimension of a few tens of nm) activity. Moreover, beyond biological applications, small-sized NDs could be of interest also in different contexts, such as for quantum gravity effects’ detection, as suggested in ref (69) or to be conjugated with quantum imaging techniques.^70^

Overall, our results demonstrate how the reported protocol can be effectively employed to obtain high-performance, small-sized NDs for quantum sensing applications, especially in the biological frame, where small nanoparticle sizes offer new and interesting opportunities.

## Supporting Information Available

Irradiation damage profile; DLS measurements in phosphate-buffered saline solution; AFM and PL image processing details; statistical test results; schematic of the different measurement conditions; and representative ODMR spectra from the different samples (PDF)
