# Supplementary material for: Effects of Thermal Oxidation and Proton Irradiation on Optically Detected Magnetic Resonance Sensitivity in Sub-100 nm Nanodiamonds
Source: ACS Appl Mater Interfaces. 2025 Mar 30;17(14):21589–600. doi: 10.1021/acsami.4c08780 (PMC11986899; doi:10.1021/acsami.4c08780)
Supplement: Supplementary file 1 — am4c08780_si_001.pdf [file am4c08780_si_001.pdf]

## Supporting Information

### Effects of thermal oxidation and proton irradiation on ODMR sensitivity in sub-100 nm nanodiamonds

Pietro Aprà [1,a], Gabriele Zanelli [1,b,c], Elena Losero*[b], Nour-Hanne Amine [a,c,d], Greta Andrini [a], Mario Barozzi [e], Ettore Bernardi [b], Adam Britel [a,c,d], Roberto Canteri [e], Ivo Pietro Degiovanni [b], Lorenzo Mino [d,f], Ekaterina Moreva [b], Paolo Olivero [a,c,d], Elisa Redolfi [a,b,c], Claudia Stella [b,g], Sofia Sturari [a,c,d], Paolo Traina [b], Veronica Varzi [a,c,d], Marco Genovese [a,b], Federico Picollo [a,c,d]

[1]*co-first authors*
**Corresponding author e-mail: e.losero@inrim.it*

a. National Institute for Nuclear Physics (Section of Torino), Via P. Giuria 1, 10125, Torino, Italy

b. Istituto Nazionale di Ricerca Metrologica, Strada delle Cacce 91, Torino, 10135, Italy

c. Physics Department, University of Torino, Via P. Giuria 1, 10125 Torino, Italy

d. NIS Inter-Departmental Centre, Via G. Quarello 15/a, 10135 Torino, Italy

e. Bruno Kessler Foundation, Center for Sensors and Devices, Via Sommarive 18, I-38123 Povo, Trento, Italy

f. Chemistry Department, University of Torino, Via P. Giuria 7, 10125 Torino, Italy

g. Politecnico di Torino, Corso Castelfidardo 39, 10129, Torino, Italy

## Damage profile

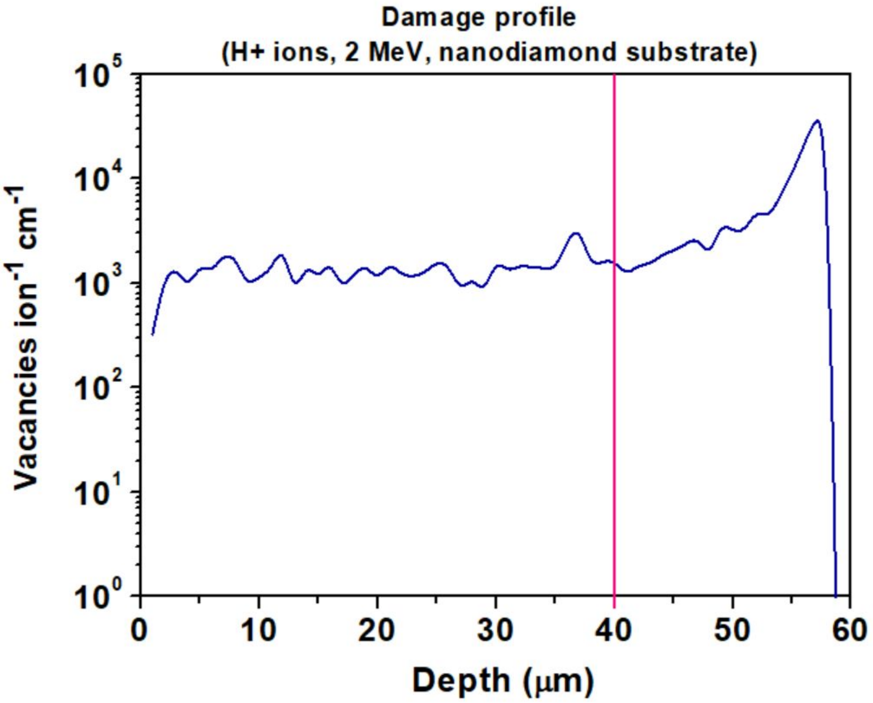


*Figure S1: linear vacancy density per ion in the NDs substrate as a function of the depth, showing that a constant damage profile is guaranteed down to 40 μm.*

## DLS in PBS solution

To investigate more in detail NDs hydrodynamic behavior, DLS measurements were performed also in phosphate-buffered saline (PBS) and the results are presented in Figure S2. Data reveal that annealed NDs display a high aggregation tendency also in this case, similarly to what is observed in water. On the other hand, oxidized NDs retain their good dispersibility in PBS. These outcomes show that the differences among the different samples are not linked to solvent-specific effects, but rather originate mainly from the hydrophobic or hydrophilic nature of the NDs surface.

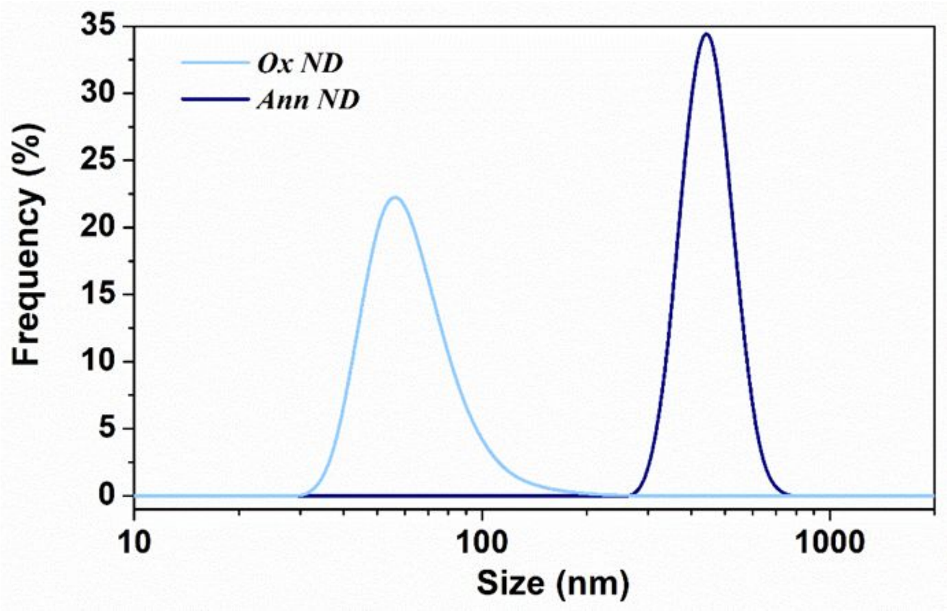


*Figure S2: Size of NDs in PBS assessed by DLS measurements (NDs concentration = 10 µg ml$^{-1}$).*

## AFM image processing

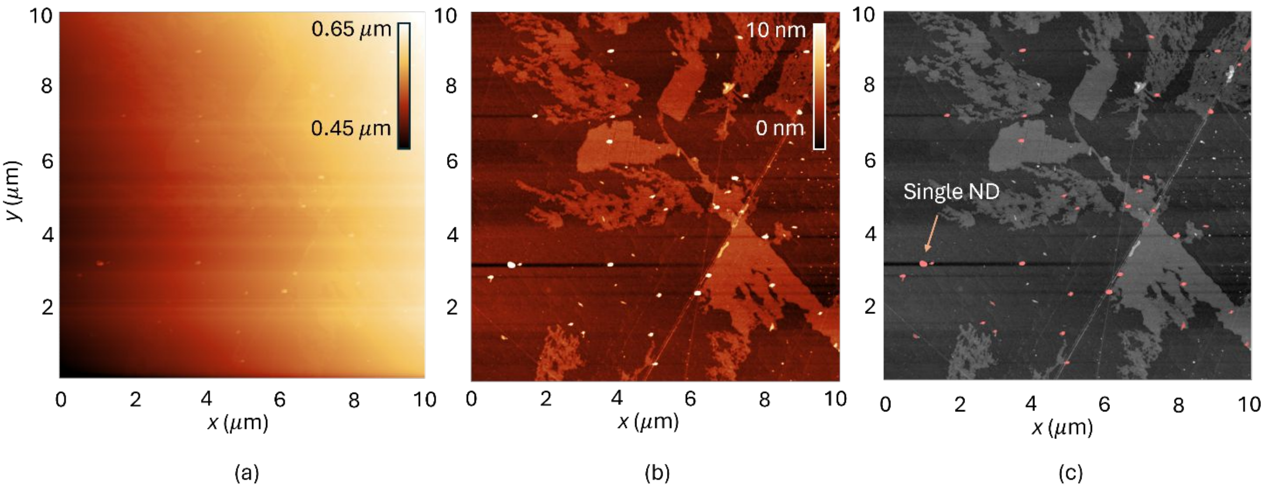


*Figure S3: AFM images processing: (a) raw image (b) image after flattening (c) identification of single NDs. NDs are identified setting an adequate threshold and filtering for the area, in order to not include in the subsequent analysis clusters and isolated spikes.*

## PL images processing

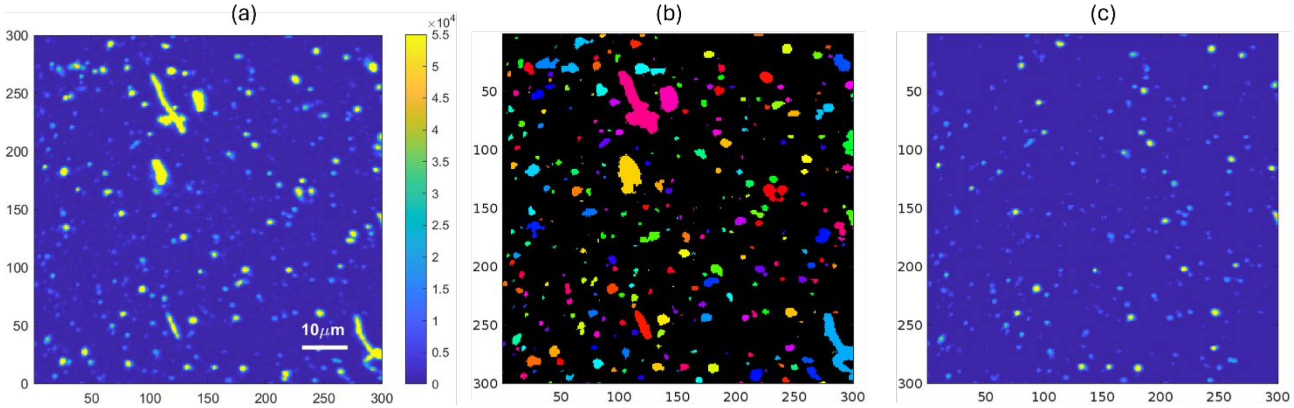


*Figure S4: PL images processing (a) PL image after background removal (b) identified objects (c) final PL image, after big cluster subtraction.*

The PL images processing is performed in Matlab and can be summarized in the following steps:

1- Background removal (see Fig. S4(a) as an example)
2- Photoluminescent object identification, as object presenting PL higher than 5 times the background noise. The binary image presenting each identified object with a different color is reported in Fig. S2(b). For each sample a number of identified objects greater than 300 is obtained.
3- Photoluminescent object filtering, according to their area. Only objects presenting an area of the order of the point-spread-function of our optical system are considered. In this way only single NDs, or small aggregates below the spatial resolution, are included in our analysis. The filtered image is reported in Fig. S2(c).
4- From each identified object many values of interest can be extracted. Among them, the max value of PL, as it is discussed in the main text.

## Statistics tests

| | $Ox^{low}$-$ND$ | $Ox^{high}$-$ND$ | $Irr^{low}$-$ND$ | $Irr^{high}$-$ND$ |
|---|---|---|---|---|
| $Ox^{low}$-$ND$ | 1 | 1 | 0.9958 | 9.6 e-4 |
| $Ox^{high}$-$ND$ | 1 | 1 | 0.9541 | 4.3 e-4 |
| $Irr^{low}$-$ND$ | 0.9958 | 0.9541 | 1 | 1.7 e-2 |
| $Irr^{high}$-$ND$ | 9.6 e-4 | 4.3 e-4 | 1.7 e-2 | 1 |

*Table S 1:* Kolmogorov-Smirnov test (comparison of PL distribution in the different samples).

| **Contrast** | $Ox^{low}$-$ND$ | $Ox^{high}$-$ND$ | $Irr^{low}$-$ND$ | $Irr^{high}$-$ND$ |
|---|---|---|---|---|
| $Ox^{low}$-$ND$ | 1 | 0.7927 | 0.0049 | 0.0799 |
| $Ox^{high}$-$ND$ | 0.7927 | 1 | 0.0058 | 0.0279 |
| $Irr^{low}$-$ND$ | 0.0049 | 0.0058 | 1 | 0.0011 |
| $Irr^{high}$-$ND$ | 0.0799 | 0.0279 | 0.0011 | 1 |

*Table S 2:* T-test (comparison of Contrast among the different samples)

| **FWHM** | $Ox^{low}$-$ND$ | $Ox^{high}$-$ND$ | $Irr^{low}$-$ND$ | $Irr^{high}$-$ND$ |
|---|---|---|---|---|
| $Ox^{low}$-$ND$ | 1 | 0.1119 | 0.0043 | 0.2899 |
| $Ox^{high}$-$ND$ | 0.1119 | 1 | 0.0272 | 0.5203 |
| $Irr^{low}$-$ND$ | 0.0043 | 0.0272 | 1 | 0.0134 |
| $Irr^{high}$-$ND$ | 0.2899 | 0.5203 | 0.0134 | 1 |

*Table S 3:* T-test (comparison of FWHM among the different samples)

| **Sensitivity** | $Ox^{low}$-$ND$ | $Ox^{high}$-$ND$ | $Irr^{low}$-$ND$ | $Irr^{high}$-$ND$ |
|---|---|---|---|---|
| $Ox^{low}$-$ND$ | 1 | 0.5619 | 0.2934 | 0.5351 |
| $Ox^{high}$-$ND$ | 0.5619 | 1 | 0.0531 | 0.1337 |
| $Irr^{low}$-$ND$ | 0.2934 | 0.0531 | 1 | 0.3252 |
| $Irr^{high}$-$ND$ | 0.5351 | 0.1337 | 0.3252 | 1 |

*Table S 4:* T-test (comparison of Sensitivity among the different samples)

## Schematic of the different measurements conditions

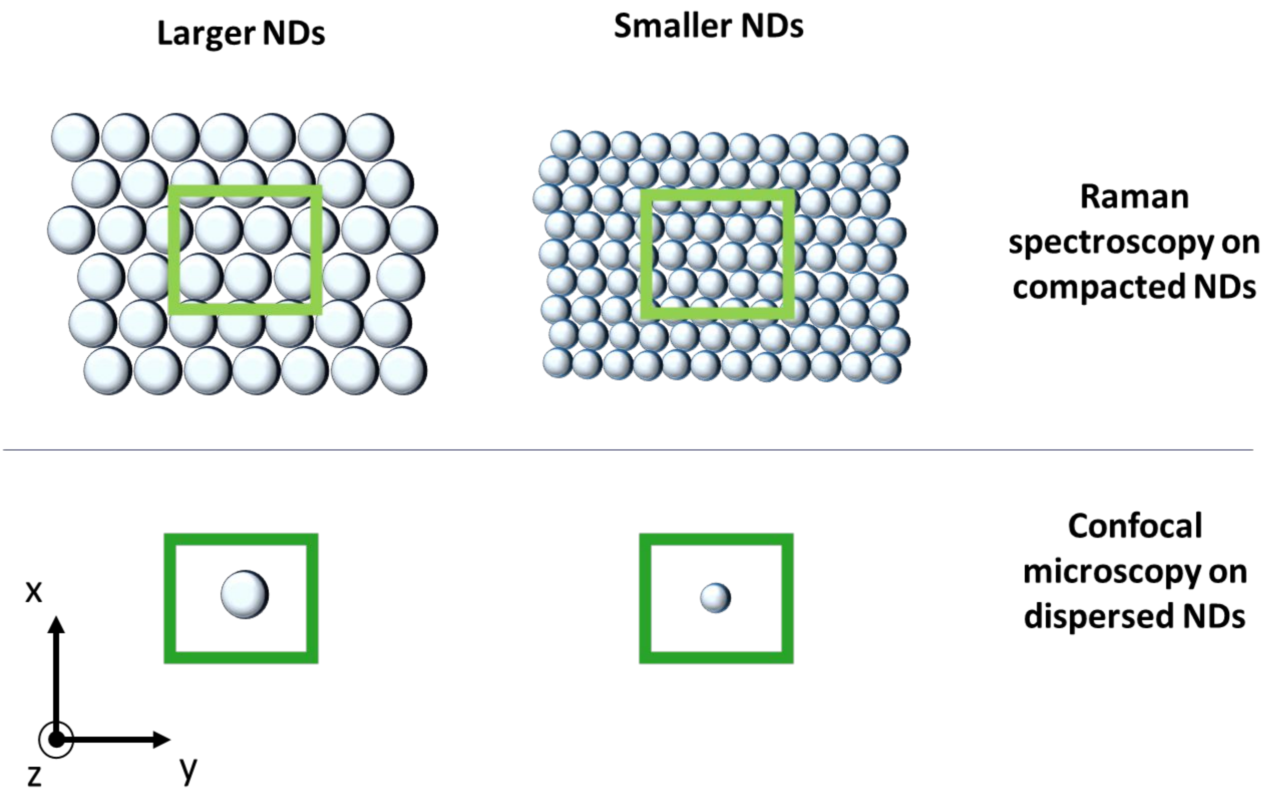


*Figure S5. Comparison between the two different PL measurements conditions used in this work. Top line: compacted NDs (using the Raman spectrometer, results reported in Figure 4). Bottom line: dispersed NDs (using the confocal microscope setup, results reported in Figure 6 and Table 1). The impact of the NDs size (right vs left column) is different with the two techniques (see text for more details).*

## Representative ODMR spectra after different treatments

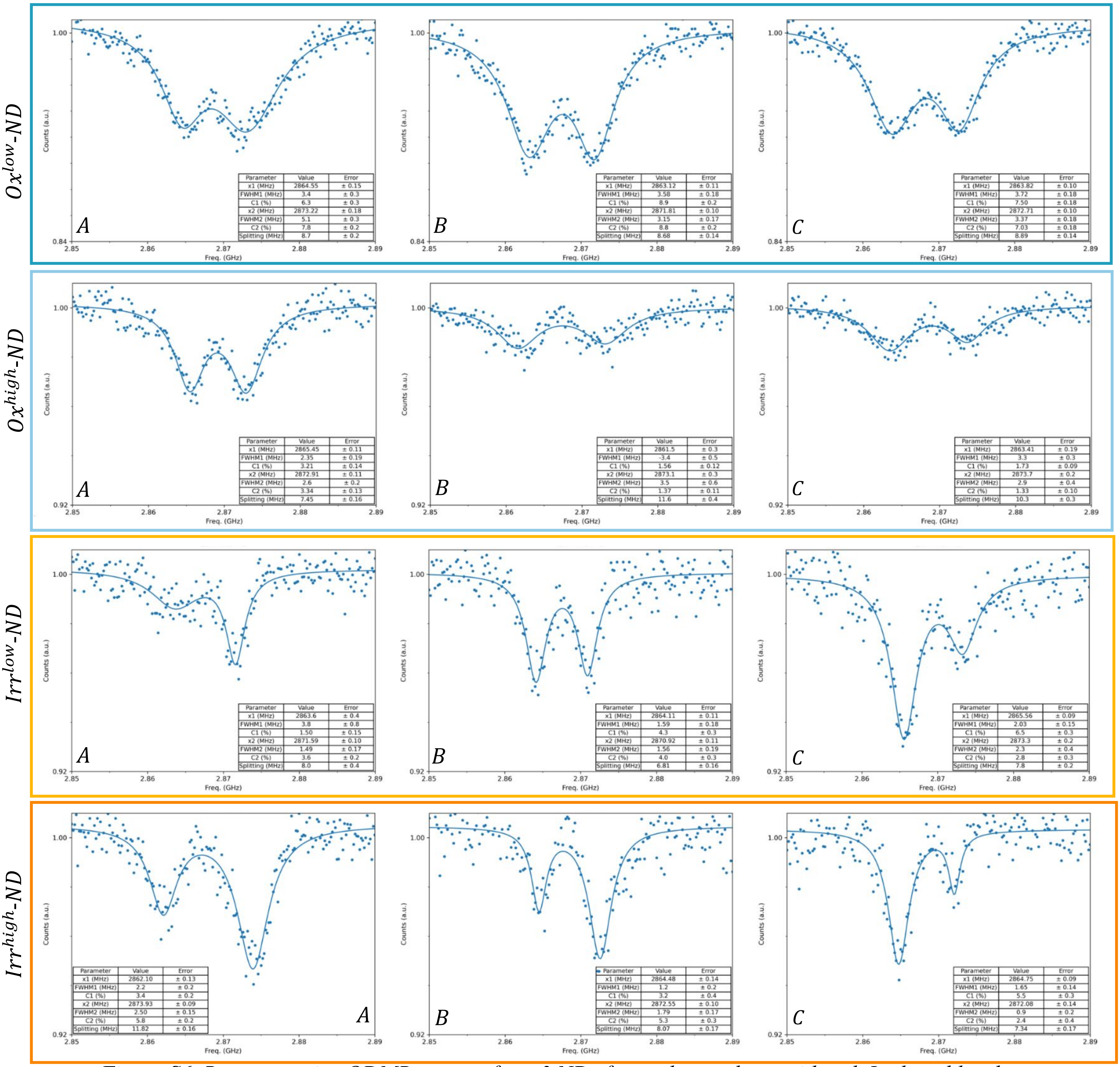


*Figure S6. Representative ODMR spectra from 3 NDs for each sample considered. In the tables the values of deeps position, FWHM, contrast and deeps splitting (with the respective uncertainties) are reported. The increased strain in the irradiated samples induces asymmetry in the spectra. This is due to the strain-induced creation of more complex spin-superposition states, which are not equally driven by the linearly polarized MW produced in our current set-up.*
